# Dysregulated Inflammation During Obesity: Driving Disease Severity in Influenza Virus and SARS-CoV-2 Infections

**DOI:** 10.3389/fimmu.2021.770066

**Published:** 2021-10-28

**Authors:** Katina D. Hulme, Ellesandra C. Noye, Kirsty R. Short, Larisa I. Labzin

**Affiliations:** ^1^ School of Chemistry and Molecular Biosciences, The University of Queensland, Brisbane, QLD, Australia; ^2^ Australian Infectious Diseases Research Centre, The University of Queensland, Brisbane, QLD, Australia; ^3^ Institute for Molecular Bioscience, The University of Queensland, Brisbane, QLD, Australia

**Keywords:** obesity, inflammation, influenza virus, SARS-CoV-2, trained immunity, cytokine

## Abstract

Acute inflammation is a critical host defense response during viral infection. When dysregulated, inflammation drives immunopathology and tissue damage. Excessive, damaging inflammation is a hallmark of both pandemic influenza A virus (IAV) infections and Severe Acute Respiratory Syndrome-Coronavirus-2 (SARS-CoV-2) infections. Chronic, low-grade inflammation is also a feature of obesity. In recent years, obesity has been recognized as a growing pandemic with significant mortality and associated costs. Obesity is also an independent risk factor for increased disease severity and death during both IAV and SARS-CoV-2 infection. This review focuses on the effect of obesity on the inflammatory response in the context of viral respiratory infections and how this leads to increased viral pathology. Here, we will review the fundamentals of inflammation, how it is initiated in IAV and SARS-CoV-2 infection and its link to disease severity. We will examine how obesity drives chronic inflammation and trained immunity and how these impact the immune response to IAV and SARS-CoV-2. Finally, we review both medical and non-medical interventions for obesity, how they impact on the inflammatory response and how they could be used to prevent disease severity in obese patients. As projections of global obesity numbers show no sign of slowing down, future pandemic preparedness will require us to consider the metabolic health of the population. Furthermore, if weight-loss alone is insufficient to reduce the risk of increased respiratory virus-related mortality, closer attention must be paid to a patient’s history of health, and new therapeutic options identified.

## Introduction

The prevalence of obesity has nearly tripled since 1975 (1, 2). By 2030 it is predicted that 40% of the world’s population will be overweight (Body Mass Index, BMI > 25 kg/m^2^) and 20% obese (BMI > 30 kg/m^2^) (3, 4). Obesity is a strong risk factor for many non-communicable diseases including: metabolic disorders (e.g., diabetes mellitus), pulmonary diseases (e.g., obesity hypoventilation syndrome), cardiovascular diseases (e.g. stoke and myocardial infarction), digestive diseases (e.g. gallstones and non-alcoholic fatty liver disease), and cancers (notably thyroid, colon and renal cancers) (4, 5). There are a number of factors that confer obesity risk, including dietary choices, socioeconomic status, sedentary lifestyle, and genetics (6). While, the exact drivers of obesity remain contentious, obesity is more than energy intake simply outweighing energy expenditure (6).

The relationship between obesity and respiratory viral disease came to prominence during the 2009 ‘swine flu’ pandemic (7). Influenza viruses are negative-sense, segmented, single stranded RNA viruses (Orthomyxoviridae family) (8). Of the four subtypes of influenza (A, B, C, D), influenza A viruses (IAVs) are the most predominant in humans, birds and other animal species. IAVs are classified by the surface glycoproteins hemagglutinin (HA) and neuraminidase (NA), of which eighteen and nine types have been isolated and identified respectively (9). Due to a low fidelity RNA polymerase, influenza virus rapidly evolves with changes primarily in the HA and NA proteins. This *antigenic drift* makes IAV adept at escaping established humoral immunity, acquired either through natural infection or vaccination. The segmented IAV genome allows for reassortment of segments from different IAV strains in a susceptible animal host, and this rapid *antigenic shift* can lead to new IAV pandemics, such as the April 2009 ‘swine flu’ H1N1. While seasonal H1N1s had been circulating previously, this new pandemic H1N1 strain caused up to 575,400 deaths in the first year of the pandemic and outbreaks have continued to cause serious illness and mortality (10, 11).

SARS-CoV-2 is a positive sense, single-stranded RNA enveloped virus (β coronavirus family) (12), which binds to the angiotensin-converting-enzyme 2 (ACE2) receptor for entry into host cells (13). As of September 2021, there have been over 232 million recorded cases of COVID-19, the disease caused by severe SARS-CoV-2 infection, with over 4.7 million recorded deaths worldwide (14). The emergence of multiple variants with apparent increased transmissibility and potential for evasion of vaccine-induced immunity poses an ongoing concern during this pandemic (15). Acute COVID-19 can range from asymptomatic to fatal disease, with symptoms including fever, myalgia, headache, respiratory symptoms, loss of taste and smell, cardiovascular complications and gastrointestinal symptoms in addition to pulmonary complications (16). These symptoms may persist for more than 4 weeks post the initial diagnosis, leading to a condition widely referred to as long COVID. Symptoms associated with long COVID include, fatigue, breathlessness, cardiac complications and neurological disease (17). The prevalence and causes of long COVID have yet to be defined.

Independent of other co-morbidities or risk factors, individuals with obesity were at higher risk of death due to IAV infection during the 2009 pandemic (7). Even when influenza vaccination is accounted for, obese adults are still twice as likely to develop influenza or influenza-like illness compared to healthy-weight adults (18). Accordingly, obese mice infected with IAV have a 6-fold increase in mortality compared to their non-obese counterparts (19), and obesity has been shown to increase the cardiovascular complications associated with IAV (20). Obesity has been reported as an independent risk factor for respiratory failure and invasive mechanical ventilation, intensive care unit (ICU) admission and death among patients hospitalized with SARS-CoV-2 infections (21–23). While obesity is a clear risk factor for respiratory viral infections, it also correlates with increased disease severity during other infections, including *Mycobacterium tuberculosis*, *Helicobacter pylori*, coxsackievirus, and encephalomyocarditis virus (24–28). While the pathogens differ, these studies suggest that a dysregulated immune response in the obese host fails to clear the pathogen and/or drives immune mediated tissue damage. For IAV and SARS-CoV-2, this may result in increased viral shedding in obese individuals (29, 30). Obesity may also promote emergence of more virulent variants of IAV (31). Obese individuals have higher ACE2 in their bronchial epithelial cells (32), suggesting increased opportunity for SARS-CoV-2 infection and replication. Experimentally, obesity increases the severity and associated mortality of secondary bacterial infections following influenza virus infection, regardless of vaccination status (33). As of yet, there are no comprehensive clinical studies to confirm these findings in humans. Intriguingly, increased BMI correlates with better outcomes (decreased case fatality rates) in bacterial respiratory infections alone, recalling the phenomenon of the ‘obesity paradox’ (34–36). The underlying mechanism for this increased protection against bacterial infection in obese patients is unknown.

In this review, we explore the hypothesis that obesity dysregulates the baseline inflammatory response, thus dysregulating innate inflammatory responses to IAV and SARS-CoV-2 infection and thereby increasing disease severity.

### Inflammation in Host Defense and Homeostasis

An effective immune response is the key to clearing any infection, and the timing, magnitude and composition of this response must be tightly controlled. A weak and delayed immune response may fail to eliminate the offending pathogen whilst an excessive and prolonged immune response can cause tissue damage and immunopathology. Failure to either eliminate or tolerate the pathogen results in a loss of tissue function, leading to disease (37). As the innate inflammatory response precedes and ultimately shapes the ensuing adaptive response (38), in this review we will focus on the regulation of innate immunity.

Acute inflammation is the body’s initial response to infection and/or injury (39), resulting in an influx of immune cells to the site of infection or tissue damage (40). This is mediated by the *innate immune* system, so named because in contrast to the *adaptive immune* system, the receptors and mediators are germline encoded. The function of the innate immune/inflammatory response during IAV or SARS-CoV-2 infection is to limit/eliminate the virus, clear dead cells, and restore tissue function. If this initial innate response is insufficient to contain the virus, then inflammation acts as an alarm to initiate, prime and shape an adaptive immune response.

In contrast, the purpose of chronic, low-grade inflammation, often known as ‘meta-inflammation’, is not so clear. More recent thinking posits inflammation itself as part of the natural process to restore tissue homeostasis (39), when other feedback loops have failed. Thus, meta-inflammation is triggered by persistent disruptions to homeostasis, e.g., nutrient availability, oxygen levels, cell number, or potentially chronic or unresolved infections. Unlike during acute inflammation, chronic inflammation is not necessarily accompanied by the tell-tale heat, swelling, pain and redness associated with acute inflammation, and systemic inflammatory indicators, such as C-Reactive Protein (CRP), are only minimally elevated. Importantly, obesity is associated with meta-inflammation (41).

An emerging aspect of the inflammatory response is the induction of tolerance mechanisms – to repair tissue damage during acute pathogen insult or to return a dysregulated system to homeostasis. Host defense is an energy intense process: leukocyte proliferation and synthesis of cytokines and other effector molecules requires glucose and glutamine availability (42). Cytokines, hormones, adipokines, mitokines, matrikines, all act on cells to induce changes in function and metabolism, as required for host defense and tolerance (42, 43). Tolerance can be local: in the lung this is mediated by the structural cells (e.g. mesenchymal cells, fibroblasts, smooth muscle cells), which remodel the extracellular matrix, produce growth factors alongside cytokines and chemokines and maintain stem cell niches as recently reviewed by Flerlage et al. (44). This is integral for promoting epithelial repair and a return to tissue function. Additionally, systemic release of these cytokines and hormones can affect overall organismal tolerance, particularly by affecting metabolism. The complex and intertwined relationship between the immune system and metabolism has been reviewed recently (42, 45).

### Functions of Inflammatory Mediators in Host Defense

Inflammation is essentially cell to cell communication, facilitated by small soluble proteins such as cytokines, chemokines, eicosanoids, and other lipid and peptide mediators. While cytokines are produced during all stages of an immune response, by innate, adaptive, and even non-immune cells (e.g., epithelial cells), the pro-inflammatory triumvirate of Interleukin (IL)-1β, IL-6 and Tumor Necrosis Factor (TNF) are amongst the first and most potent cytokines released during a viral infection. IL-1β has pleiotropic actions in host defense: acting upon the nervous system to trigger fever and appetite loss, generating more immune cells in the bone marrow (hematopoiesis), increasing delivery of immune cells and mediators to the site of infection *via* the vasculature (vasodilation, angiogenesis) and activation of antibody and T cell responses (38, 46). IL-6 promotes platelet release, Cytotoxic T Lymphocyte (CTL) differentiation and antibody production, and induction of the ‘acute phase response’ (increasing serum CRP and serum amyloid A) (47). TNF variably promotes cell differentiation (e.g., T cells) or cell death (e.g., infected cells) and increases vascular permeability and leukocyte extravasation into tissue (48, 49). The ‘flu like’ symptoms experienced during infection, or during vaccination, result from the systemic actions of these cytokines.

Of all the cytokines induced during a viral infection, the interferons (IFNs) are perhaps the best known and studied. The canonical anti-viral cytokines, IFNs signal through their cognate receptors and induce expression of up to 400 interferon stimulated genes (ISGs) (50). ISGs are critical for cell *intrinsic* defense against viruses: they encode typical restriction factors that target various stages of a viral life cycle (e.g., entry: IFITM3; nuclear import: MX1, MX2; mRNA synthesis: APOBECs, Protein synthesis: PKR; Replication: OAS1; Egress: Tetherin) (50). Additionally, IFNs can act on immune cells to further amplify the inflammatory response e.g., by inducing expression of the chemokine C-X-C motif chemokine ligand 10 (CXCL10), which activates dendritic cells, enhances cross presentation, and promotes the enhancement and maturation of T and B cell responses. Paradoxically, IFNs can also strongly induce IL-10 expression, the best known of the ‘anti-inflammatory’ cytokines (51).

### Specific Receptors and Pathways Driving Inflammation in IAV Infection

Significant progress has been made in identifying how IAV triggers inflammation. Most cells express pattern recognition receptors (PRRs) to detect *intrinsic* viral infection (52). The endosomal Toll Like Receptors (TLRs) 3, 7 and 8 could all potentially sense IAV-derived RNAs of incoming IAV virions, though only TLR3 is reported to be expressed in airway epithelial cells (53). TLR7 in plasmacytoid dendritic cells (pDCs) recognizes the ssRNA IAV genome, resulting in IFN-α and pro-inflammatory cytokine release (54, 55). Recent evidence suggests that airway epithelial TLR3 is responsible for driving IFN-β production in response to IAV infection, as determined by genetic mapping of TLR3 associated mutations in children who acquire severe IAV-induced Acute Respiratory Distress Syndrome (ARDS) (56). Post fusion, viral ribonucleoproteins (vRNPs) are trafficked to the nucleus for replication. These incoming vRNPs can potentially be sensed by Z-DNA-binding protein 1 (ZBP1) to trigger NLRP3 inflammasome activation (57). The inflammasome is a multimeric cytosolic signaling platform that cleaves IL-1β and IL-18 into their mature, bioactive forms and initiates an inflammatory cell death: pyropotosis (58). NLRP3 inflammasome activation is myeloid restricted, however whether and how epithelial inflammasomes are also activated in response to IAV remains contentious (59, 60). Once the IAV vRNPs reach the nucleus, IAV replicates and in the process generates Z-RNAs which are sensed by nuclear ZBP1 to drive necroptosis (61), another form of inflammatory cell death. Replicating IAV RNA intermediates are also sensed by Retinoic-acid Inducible Gene I (RIG-I), presumably in the nucleus, to drive Type I and III IFN and pro-inflammatory cytokine expression (62). Newly synthesized vRNPs can also be sensed by RIG-I in the cytosol, where RIG-I, along with its counterpart dsRNA sensing receptor melanoma differentiation associated protein 5 MDA-5, is typically expressed (52). RIG-I and MDA-5 signal *via* the adaptor protein Mitochondrial antiviral-signaling protein (MAVS), to activate the canonical pro-inflammatory transcription factor Nuclear Factor kappa B (NF-κB) (responsible for IL-1β, IL-6 and TNF expression among many others) and the Interferon Regulatory Factors (IRFs), especially IRF3 which drives Type I and III IFN expression (52). How nuclear RIG-I signals to cytoplasmic MAVS during infection remains to be elucidated. During new viral protein synthesis, the IAV M2 ion channel activates the NLRP3 inflammasome (63) to cleave IL-1β, though whether this also triggers pyroptotic cell death is unclear.

Cells also express PRRs on their cell surface or in endosomes to detect cell *extrinsic* danger, such as infection or damage of neighboring cells. Best known among these are the TLRs and the C-type Lectin Receptors (CLRs), which are able to detect both pathogen-associated molecular patterns (PAMPs, e.g., viral RNA, bacterial lipopolysaccharides (LPS), carbohydrate moieties on viral proteins) and host danger associated molecular patterns (DAMPS: e.g., High Mobility Group Box 1 Protein: HMGB1). Endosomal TLR3, 7 and 8 in neighboring, bystander epithelial or immune cells can also recognize extracellular viral RNAs released from infected cells or revealed upon phagocytosis of dying infected cells. Surface expressed TLR4 may also amplify the inflammatory response to IAV infection by sensing DAMPS proposed to include HMGB1 and oxidized phospholipids (64). Endothelial cells also contribute to the IAV-induced cytokine storm, releasing pro-inflammatory cytokines downstream of Sphingosine 1 receptor activation (65, 66). Epithelial cells, macrophages (both alveolar and infiltrating) and endothelial cells may all contribute to the release of pro-inflammatory cytokines and anti-viral IFNs during IAV infection, though the specific contribution of each of these cell types, and their signaling pathways to host defense in IAV infection remains to be determined.

### Specific Receptors and Pathways Driving Inflammation in SARS-CoV-2 Infection

**Figure 1:**
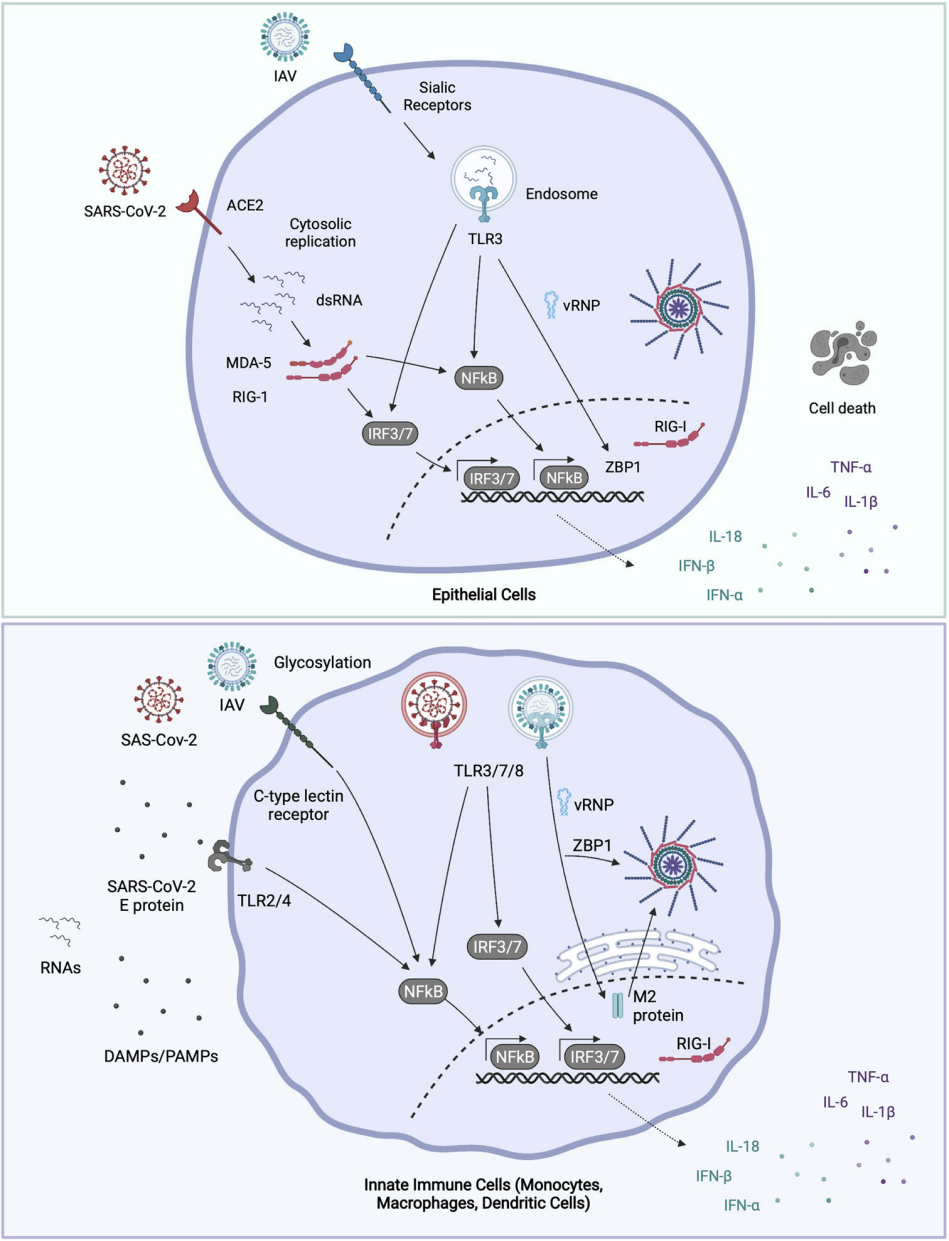
Molecular sensing of IAV and SARS-CoV-2. IAV binds to surface α2, 6-linked sialic acid (α2,6-SA) receptors on airway epithelial cells, and some macrophages, whereupon it’s internalized into endosomes (76). Incoming virions may be sensed by TLR3 (epithelial cells) or by TLR3, 7 and 8 (macrophages and dendritic cells). TLR engagement leads to NFκB and IRF transcription factor activation and pro-inflammatory and anti-viral gene expression. Post fusion, viral RNA bound by nucleoprotein (vRNPs) are released into the cytosol. Once in the cytosol, vRNPs are trafficked to the nucleus where IAV replicates. IAV replication products are sensed by nuclear RIG-I or ZBP1, triggering gene expression or cell death respectively. ZBP1 may also intercept vRNAs in the cytosol and trigger inflammasome activation. Newly synthesized IAV M2 protein can trigger NLRP3 inflammasome activation and IL-1β release. SARS-CoV-2 enters airway epithelial cells *via* ACE2, and after fusion at or near the plasma membrane, the viral genome is released into the cytosol. The dsRNA intermediates generated during SARS-CoV-2 replication are sensed by both RIG-I and MDA-5 in the cytosol, leading to NFκB and IRF3 activation. Dendritic cells and macrophages do not appear to be productively infected with SARS-CoV-2 (71). Though whether they can still take up virus, and signal from endosomal TLRs isn’t known. TLR7 in plasmacytoid dendritic cells recognises either virions or ssRNAs from SARS-CoV-2. Extracellular viral PAMPS and DAMPS released from neighbouring infected cells can also be sensed by surface and endosomal TLRs [e.g., SARS-CoV-2 envelope ‘E’ protein: TLR2 (74)], driving NFkB activation. Glycosylated viral proteins can further be detected by CLRs expressed on macrophages, which drives NFκB activation (77). Figure created with BioRender.com.

Rapid research developments in the last year have helped elucidate some of the pathways by which inflammation is triggered in SARS-CoV-2 infection. As with IAV, innate sensing of SARS-CoV-2 is determined by its infectious cycle. In airway epithelial cells expressing the receptor ACE2 and the protease transmembrane serine protease 2 (TMPRSS2), SARS-CoV-2 fusion and entry occurs at or near the cell surface. Translation and replication of the SARS-CoV-2 genome subsequently occurs in the cytosol (67). In the absence of TMPRSS2, the virus is internalized into endosomes, where the Spike protein can be cleaved by cathepsins, promoting fusion from this compartment (68). Presumably, this would also promote detection by endosomal TLRs, and may result in enhanced pro-inflammatory signaling in cells that express ACE2 but lack TMPRSS2. The dsRNA intermediates of the replicating SARS-CoV-2 genome are sensed by both RIG-I and MDA-5 (69). There is significant evidence of inflammasome activation in severe COVID-19 (70), though the cellular and molecular source of this activation is controversial, as conventional inflammasome activating cells (e.g., macrophages, dendritic cells) are not infected (71), precluding cell intrinsic inflammasome activation by SARS-CoV-2. Recently the epithelial NLRP1 inflammasome was identified as a sensor for cytosolic dsRNA (72). Whether this is activated in response to SARS-CoV-2 infection and drives disease remains to be determined. Nevertheless, cell *extrinsic* sensing of SARS-CoV-2 also drives pro-inflammatory and anti-viral cytokine release. As with IAV, SARS-CoV-2 virions or SARS-CoV-2 derived ssRNAs are sensed by TLR7 in pDCs, stimulating critical IFN-α production. As such, genetic deficiency of TLR7 is associated with severe COVID-19 phenotypes (73). TLR2, which is highly expressed on myeloid cells, can detect the SARS-CoV-2 envelope (‘E’) protein, and drive pro-inflammatory gene expression (74), while C-type Lectins on the surface of macrophages and other myeloid cells detect the glycans attached to the SARS-CoV-2 Spike (75). TLR2 and CLRs primarily drive NF-κB dependent gene expression and so signaling from these cell surface myeloid receptors may skew the cytokine balance in COVID-19 from anti-viral to pro-inflammatory. An overview of the innate sensing and signalling pathways implicated in IAV and SARS-CoV-2 infection are provided in **Figure 1**.

### Detrimental Inflammation in IAV and SARS-CoV-2 Infection

Expression of pro-inflammatory cytokines (e.g., TNF, IL-6, IL-1β), are all critical in defense against both IAV and SARS-CoV-2. However, in excess or when prolonged, these cytokines all enhance disease severity. Dysregulated or hyperinflammatory immunopathology are now recognized as key indicators and drivers of disease severity in both pandemic IAV and COVID-19 (78–83). Raised levels of pro-inflammatory cytokines and chemokines, such as IL-1β, IL-6 and TNF have been identified as indicators of lung injury following IAV infection (84–87). Newly recruited monocytes, macrophages, and neutrophils entering the IAV-infected and -inflamed lung secrete further pro-inflammatory cytokines, further amplifying the response (88). The consequence of this inflammatory loop is tissue damage, including diffuse alveolar damage, hyaline membrane formation, fibrotic healing, capillary damage and immunopathologic injury, often leading to ARDS (89, 90). The virulence of the 1918 pandemic IAV is attributed to its ability to cause a hyper inflammatory response (91), while highly pathogenic avian IAVs also trigger excessive cytokine release in cell and animal models compared to seasonal strains (92–95). Targeting these inflammatory signaling pathways therapeutically in IAV infection remains challenging as they are critical for early host defense. For example, NLRP3 inflammasome activation and IL-1β responses are critical for developing a functional adaptive response to IAV: mice deficient in inflammasome components succumb more quickly to IAV infection (96, 97). Similarly, treating mice with the small molecule NLRP3 inhibitor MCC950 prior to IAV infection resulted in greater IAV lethality (98). Promisingly, inhibiting NLRP3 at the peak of IAV disease severity (e.g., Day 8 and 9) improved disease outcomes in mice, suggesting that therapeutic timing of any anti-inflammatory treatment is critical. In IAV infections, this hyperinflammatory response is succeeded by a tolerized or immune-suppressed state, during which secondary (and often fatal) bacterial infections can take hold (37). How these anti-inflammatory drugs might dampen the IAV-induced cytokine storm, without increasing susceptibility to secondary bacterial infection remains a limitation to their use in the clinic.

The presentation of SARS-CoV-2 infection in patients ranges from a mild respiratory tract infection to a serious and systemic inflammatory response (99, 100). Some patients with SARS-CoV-2 experience dysregulated cytokine responses characteristic of a cytokine storm with elevated levels of cytokines such as IL-6, IL-10, IL-1β, IL-18, TNF, IFNs, and CXCL10 (99). Similarly, patients with severe COVID-19 have an influx of inflammatory monocytes and macrophages into their lungs, which correlates with increased cytokine expression (101). Vascular dysfunction, clotting and increased risk of stroke and heart attack are a further pathological feature of COVID-19. SARS-CoV-2 does not directly infect endothelial cells, instead vascular dysfunction also appears to be driven by the enhanced inflammatory state (102).

The role that these pro-inflammatory cytokines play in driving severe disease in COVID-19 has been confirmed by the relative success of the broad-spectrum immunosuppressant dexamethasone (103). Similarly, clinical trials for the IL-1α/β blockers Anakinra (104) and Canakinumab (105) have shown promise in improving COVID-19 outcomes, while the IL-6 antagonist Tocilizumab is approved by the FDA under an emergency use authorization for combination use with dexamethasone for added benefit (106). Similarly, while defective early IFN responses are associated with COVID-19 disease severity (107), when IFNs are present in excess or for a prolonged period, they can disrupt epithelial repair and correlate with severe COVID-19 (108). Thus, the timing, magnitude, and balance of these cytokine responses are critical to successfully eliminating the virus without triggering collateral tissue damage. A summary of studies elucidating the role of these cytokines in IAV and SARS-CoV-2 infection is detailed in **Table 1**.

**Table 1:** Cytokines in disease severity in Influenza and SARS-CoV-2 Infections.

Cytokine	Murine model (Influenza) in study	Influenza virus in study	Cytokine Protective/Detrimental in study	Evidence	Mechanism	Potential Role with Obesity
TNF	TNF neutralizing antibody (109).	H3N2 X31	Detrimental	Inhibition of TNF with antibodies reduced lung injury and improved survival in mice.	Pro-inflammatory recruitment and activation of macrophages, NK cells, T cells, and antigen-presenting cells (110). Amplifies expression of other pro-inflammatory cytokines and chemokines in the environment (110, 111).	Enhanced lung mRNA expression and BALF concentrations in obese mice (19, 112). Thought to contribute to cytokine storm and exacerbate pathological lung injury.
	TNFR1^-/-^ mice (113).	H1N1 r1918	Detrimental	TNFR^-/-^ mice had reduced weight loss and increased survival time relative to WT mice.		
	TNF neutralizing antibody (114).	H1N1 A/PR/8/34	Detrimental	Severity of lung lesions positively correlated with bronchoalveolar TNF levels. Antibody treatment improved lesions and survivorship.		
	TNF^-/-^ mice (115).	H1N1 A/FM/1/47	Protective	TNF^-/-^ mice exhibited greater severity and pathology upon infection relative to WT mice.		
IL-6	SOCS TG mice (116).	H1N1 A/WSN/33	Detrimental	Knockdown SOCS3 mice had reduced expression of IL-6 relative to WT mice and were protected from severe IAV infection.	Regulates T-cell response, inflammation resolution, tissue remodeling and lung repair (117–119).	Excessive IL-6 is associated with poor influenza prognosis (120, 121). Obese individuals exhibit higher concentrations of IL-6 (122, 123). Excess levels thought to contribute to pathological cytokine storm (122).
	IL-6^-/-^ mice (117).	H1N1 A/WSN/33	Protective	IL-6^-/-^ mice exhibited higher severity and lethality upon infection than WT mice.		
	IL-6^-/-^ mice (119).	H1N1 A/PR/8/34	Protective	IL-6^-/-^ mice exhibited high severity and lethality upon infection than WT mice.		
IL-1β (NLRP3)	IL-1β neutralizing antibody (124).	H1N1 A/PR/8/34.	Detrimental	Neutralizing IL-1β limited inflammation in lung tissue sections.	Induces expression of pro-inflammatory genes (125, 126). Recruits T cells and neutrophils and triggers release of IL-6 and TNF by epithelial and endothelial cells (122).	Early protective role in infection. Obese mice have reduce IL-1β 3 days post infection which may contribute to increased severity (19). Later in infection, may promote pulmonary tissue pathology *via* cytokine storm (125, 127).
	IL-1β neutralizing antibody (75).	H3N2 HKx31.	Detrimental	Treatment with intranasal antibody prolonged survival and reduced inflammation in airways relative to untreated mice.		
	MCC950 (NLRP3 inhibitor) (98).	H3N2 HKx3	Detrimental and Protective	MCC950 treatment within 24 hours post infection accelerated weight loss and mortality. MCC950 treatment on day 3 and 5 improved survival and reduced lung inflammation.		
IL-18(NLRP3)	IL-18^-/-^ mice (128).	H1N1 A/PR8/34.	Protective	IL-18^-/-^ mice had increased mortality, higher pathology in lung, viral growth and infiltration of immune cells relative to WT mice.	Enhances cytokine production by CD8+ T cells and cytotoxicity of NK cells (128).	Unknown.
	IL-18^-/-^ mice (129).	H3N2 HKx31	Protective	IL-18^-/-^ mice had limited cytokine production and impaired viral clearance relative to WT mice.		
Type I IFNs	IFN-α/βR-/- mice (130).	H3N2 X31 and H1N1 A/California/04/09	Detrimental	IFN-α/βR^-/-^ mice exhibited lower morbidity and mortality, reduced lung inflammatory recruitment relative to WT mice.	Expressed by myeloid and parenchymal cells (121). Triggers expression of interferon-stimulated genes (ISGs) in nearby cells (131).	Type I IFN responses are delayed in obese mice but heightened in later stages of infection (132, 133). Enhanced IFN-α and IFN-β in later stages of infection can contribute to a cytokine storm (130, 134).
Type II IFNs	IFN-γ neutralizing antibody (135).	H1N1 pdm/09.	Detrimental	Antibody treatment improved symptoms and survival of mice by reducing acute lung injury.	Produced by NK cells in early influenza infection and T cells in later stages (136, 137). Promotes release of epithelial cytokines (138, 139).	Obese individuals have reduced release of IFN-γ from T cells in influenza infection (140).
	IFNγR^-/-^ mice (141).	H1N1 A/WSN/33 IAV.	Detrimental	IFNγR^-/-^ mice had lower inflammatory chemokine and cytokines early infection, and milder clinical symptoms and lower lung viral titers late infection.		
Type III IFNs	Il28ra1^-/-^ mice (142).	H1N1 A/PR/8/34.	Protective	Il28ra1^-/-^ mice were slightly more susceptible to influenza.	Produced by epithelial and myeloid cells (143). IFN-λ damages the lung epithelial barrier and inhibits lung repair in mice (108).	Upregulated expression of SOCS1 and SOCS2 mRNA expression in lungs of obese mice (negative regulation of Type I and III IFNs) (144). Suggests reduced Type III IFNs.
	Il28ra1^-/-^ IFNAR1^-/-^ against IFNAR1^-/-^ mice (145).	H1N1 A/HH/05/09 and B/Lee/40	Protective	Il28ra1^-/-^ IFNAR1^-/-^ mice had enhanced susceptibility and delayed viral clearance relative to IFNAR1^-/-^ mice.		
IL-17 (IL-17A and IL17F)	IL-17^-/-^ mice and anti-IL-17 antibody (146).	A/Beijing/501/09	Detrimental	IL-17^-/-^ mice had improved survival rate, body weight and lung histopathology. Treatment of WT mice with antibodies against human IL-17 improved survival rate lung histopathology, and reduced inflammation.	Produced by γδ T cells, CD4+ T helper cells, and NK cells (122).	Unknown.
IL-10	IL-10^-/-^ mice (147).	H1N1 A/PR/8/34	Detrimental	IL-10^-/-^ mice had increased survival relative to WT mice.	Produced by CD4+ and CD8+ T cells (148). Regulates other inflammatory cytokines (147, 149, 150).	Early-phase IL-10 may aggravate pulmonary inflammation, but late phase may assist recovery (150).
	IL-10^-/-^ mice (149).	H1N1 A/PR/8/34	Detrimental	IL-10^-/-^ mice had improved viral clearance and survival after infection relative to WT mice.		
	Anti-IL10 antibody mice (148).	H1N1 A/PR/8/34	Protective	Antibody treatment to block IL-10 receptor lead to lethal pulmonary inflammation and immune infiltration, accompanied by increased cytokine expression.		
**Cytokine**	**Model (SARS-Cov-2) in study**	**SARS-CoV-2 Virus in study**	**Cytokine Protective/Detrimental in study**	**Evidence**	**Mechanism**	**Potential Role with Obesity**
Type I IFNs	Human – genetic variants in loci involved in TLR3- and IRF7 dependent induction and amplification of type I IFNs (151).	SARS-CoV-2	Protective	3.5% of patients with potentially lethal COVID-19 pneumonia had genetic deficiencies in TLR3 or IRF7 induction/amplification of type I IFNs.	IFN-1 responses are impaired in patients with severe COVID-19 (152).	Unknown.

### Obesity and Inflammation

Genetics and diet are the two primary drivers of obesity. BMI and obesity may be heritable (153, 154) and this is attributed to both monogenic and polygenic traits (155). These include mutations in leptin and its receptor (LEPR) (156, 157), prohormone convertase 1 (PC1) (158), and fat mass and obesity associated (FTO) gene (155, 159). Mutations in the leptin-melanocortin signaling pathway, most of which occur in the melanocortin 4 receptor (MC4R), may account for much of the heritability of obesity (159): an estimated 1 in 200 obese people worldwide have disease-causing mutations in MC4R (160). The genetic contribution of obesity has been explored experimentally in the *ob* (mutation in the gene encoding leptin) and *db* (mutation in gene encoding leptin receptor) mouse models (6). While only 1-5% of the morbidly obese population can be attributed to a mutation related to leptin or its receptor (161, 162), these models still have use as they recapitulate the leptin resistance and attenuation of leptin signaling that occurs during diet induced obesity (163).

Global urbanization has been accompanied by a shift from diets rich in plant-based fiber to a high-fat, high-protein, calorie rich diet referred to as the ‘Western Diet’ (164). The Western Diet (WD) consists of high amounts of processed, sugar and carbohydrate rich foods, with a distinct lack of vitamins, minerals, and fiber (41). Long-term consumption of this diet results in weight gain and increased BMI: accumulation of triglycerides from carbohydrates in a WD amplifies insulin signaling, which promotes increased storage of glucose and fatty acids in adipose tissue (165, 166). Diet-induced obesity models in rodents reproduce human diets using two main forms: The WD with 49% energy from carbohydrates, or a High Fat Diet (HFD), with 45-60% of energy from fat (167–171) and are most commonly used with C57BL6/J male mice for a period of 12-20 weeks (167, 170). Both models induce weight gain, increased systemic inflammation (circulating cytokine levels in serum), increased expression of inflammatory genes in adipose tissue, and alter body composition, glucose levels, insulin levels and insulin resistance (167–172).

### Activating and Regulating Inflammatory Pathways in Obesity

Several components of the western diet can trigger inflammation directly through PRR activation (e.g., cholesterol crystals activate NLRP3 inflammasome activation) while others, such as excess fatty acids, L-carnitine and phosphatidylcholine cause dysbiosis in the gut microbiome, thereby indirectly triggering PRR activation (41). For example, a long term processed food diet increases intestinal barrier permeability, in part due to the presence of advanced glycation end-products (AGEs), which are a group of posttranslational modifications that are generated under thermal food processing (173). This barrier permeability lead to systemic circulation of LPS from commensal bacteria present in the gut. LPS triggers systemic innate immune activation, presumably *via* TLR4 or *via* the non-canonical caspase-driven inflammasomes: caspase 4/5 in humans and caspase-11 in mice (174, 175). Accordingly, treatment of the obese mice with alagebrium (an AGE inhibitor that breaks the heat–induced crosslinks) or a high starch fiber diet improved gut barrier integrity and reversed the systemic inflammation (173).

Obesity also induces remodeling and enlargement of adipose tissue to accommodate the storage of excess energy obtained from the diet (176). This is mediated by both increases in adipocyte mass and number and is accompanied by enhanced levels of immune cell infiltration and polarization toward more “inflammatory” adipose tissue macrophages. Both adipose associated immune cells (e.g., macrophages) and adipocytes release pro-inflammatory cytokines (177) and adipokines, with obesity leading to increased serum leptin (178), increased serum resistin (179), and reduced serum adiponectin (180–182). This imbalance of adipokines partly contributes to chronic systemic inflammation.

These adipokines and hormones can either amplify or suppress inflammatory responses (43). Intriguingly, metabolic changes can occur even without an increase in adipose tissue mass or BMI, rather an urban diet alone (increased intake of processed and high fat foods) increases pro-inflammatory gene expression compared to a plant and fiber based diet (183). This suggests that diet itself, rather than necessarily BMI and adipose tissue size, is enough to modulate adipokine, hormone and cytokine levels and thereby impact the inflammatory response.

Other hormones that regulate metabolism include Fibroblast Growth Factor 21 (FGF21) and Growth and Differentiation Factor 15 (GDF15). FGF21, released primarily from the liver, induces energy expenditure (184, 185), thermoregulation and maintains cardiac function under stress (184–186), whereas GDF15 supresses food intake (187). Serum levels of both FGF21 and GDF15 are increased in obesity (188), which might suggest a pathological function, however multiple studies suggest that FGF21 and GDF15 are both protective and essential for healthy metabolism as reviewed by Keipert et al. (189). Akin to leptin resistance in obesity, increased FGF21 and GDF15 levels in obesity might indicate resistance to these cytokines, and a concomitant decrease in function (189), though this remains unresolved. Both FGF21 and GDF15 are critical in host defense by promoting tolerance: in models of bacterial sepsis they induce thermoregulation, and triglyceride release respectively (190) and thus protect the heart by regulating the increased cardiac metabolic demands of infection. Consistent with this, blocking GDF signaling increased susceptibility to IAV infection in mice due to loss of hepatic sympathetic outflow and triglyceride metabolism, resulting in impaired cardiac function and maintenance of temperature, indicating that loss of GDF15 is highly detrimental in infection (190). However, whether dysregulation of FGF21 and GDF15 in obesity affects tolerance during IAV or SARS-CoV-2 infection remains to be experimentally confirmed.

### Increased Inflammation in Obese Hosts During IAV and SARS-CoV-2 Infection

Considering their critical roles in early host defense, an elevation of pro-inflammatory cytokines and chemokines in obese hosts might be expected to confer extra protection against invading viruses such as IAV and SARS-CoV-2. In contrast, during the 2009 H1N1 pandemic, severe infections were characterized by significantly higher levels of IL-6 and IL-8 (121). In particular, non-survivors had elevated levels of IL-6 compared to survivors (121). High levels of these inflammatory biomarkers are associated with the obese state (191, 192), suggesting a predisposition to severe influenza outcomes.

High levels of IL-6 and CRP are associated with the obese state (193–195) and associated with the development of ARDS in obese individuals with COVID-19 (196). Furthermore, in SARS-CoV-2 infection, BMI is positively associated with increased pulmonary inflammation and increased pro-inflammatory cytokines and metabolic markers in the serum while being negatively associated with Spike-specific IgG antibody levels (197).

### How Does Obesity Dysregulate the Inflammatory Response to IAV and SARS-CoV-2?

Inflammatory signaling is tightly regulated, with multiple layers of negative and positive feedback loops (198). Loss of negative regulators, or over stimulation of positive regulators can have profound effects on the inflammatory milieu. Obesity may affect the inflammatory response by changing the expression or function of these regulators. This can include PAMP sequestration (e.g. viral proteins by antibodies, LPS by High-Density Lipoprotein: HDL), receptor trafficking (the PRR needs to be present in the correct subcellular location to detect the PAMP or DAMP), phosphorylation, ubiquitination (199) and other post-translational modifications (200). Inducible negative regulators block signaling (e.g., Suppressor of Cytokine Signaling 3: SOCS3) while transcriptional repressors suppress pro-inflammatory gene expression. Many of these negative regulators, such as Activating Transcription Factor 3 (ATF3) are induced by PRR stimulation itself (201), Type I Interferons (202) and HDL (203). Metabolic changes downstream of PRR stimulation also drive histone modifications that have both short- and long-term effects on pro-inflammatory gene expression (204, 205). Post-transcriptionally, microRNAs and mRNA destabilizing agents serve to further titrate pro-inflammatory mRNA levels (198). Decoy receptors and cytokine antagonists (e.g. IL-1R antagonist) further restrain inflammatory signaling downstream of cytokines, while anti-inflammatory cytokines including IL-10 (206), Transforming growth factor (TGF)-β, and Type I IFNs (207) (depending on context) block pro-inflammatory signaling by inducing many of the above described negative regulators.

The inflammatory response to an infection is therefore highly dependent on the local micro-environment, which determines expression and activity of these negative regulators. For example, HDL levels are lowered in obesity, removing a critical negative regulator from the inflammatory cascade (208). Thus, single, or cumulative changes to the levels and functions of enzymes and proteins that regulate inflammation, as may occur during obesity, can hugely impact the timing, magnitude, and balance of the pro-inflammatory and anti-viral response. The effect of obesity specifically on these regulatory pathways has only been partially explored, as will be discussed in the next section, and further investigation into the effect of obesity on mechanisms of inflammatory regulation, particularly in the molecular pathways involved in IAV and SARS-CoV-2 sensing, are warranted.

### Obesity-Induced Trained Immunity

Various studies have demonstrated that monocytes from obese children and adults produce increased cytokines (notably TNF) upon *ex vivo* stimulation with TLR agonists, relative to monocytes from non-obese individuals (209–213). This heightened response is consistent with the phenomenon of ‘trained immunity’ where innate immune cells have a heightened pro-inflammatory response upon exposure to a secondary immunological stimulus (214, 215). Trained monocytes exhibit increased histone-3-lysine-27-acetylation (H2K27Ac) and H3 histone-lysine-4-trimethylation (H2K4me3) on gene promoters related to the inflammatory response and metabolism, and increased H3K4me1 in enhancer regions (216). This epigenetic memory is therefore influenced by changes in intracellular metabolism, with metabolites regulating the activity of these histone modifying enzymes (217–219). Thus, the heightened pro-inflammatory response upon secondary stimulation results from global metabolic and epigenetic reprogramming of innate immune cells by the first stimulus.

The increased pro-inflammatory phenotype of monocytes in obese patients has been associated with global increases in histone H4 acetylation (210). In addition, monocyte priming after a western-style, dislipidemia diet in non-human primates was correlated with increased H3K27 acetylation (210, 220). Monocytes from obese patients have upregulation of metabolic pathways related to inflammation, cholesterol synthesis, and glucose metabolism (221), reflective of changes observed in *in vivo* trained immunity studies. In mice, a WD induces a long-term hyper-responsive innate immune state, characteristic of trained immunity, and importantly, this persists even following weight loss (171).

Peripheral monocytes and DCs have an average lifespan of 5 days which is not long enough account for the observations of a trained phenotype years or decades after the primary stimulus (222). Strong evidence suggests this training occurs *via* modulation of bone marrow hematopoietic stem and progenitor cells (HSPCs), which then carry the relevant epigenetic changes to their daughter cells (171, 223). HSPCs express TLRs and cytokine receptors, and consequently have been shown to exhibit a trained phenotype following exposure to PAMPs and circulating cytokines (223–226), such as might occur during IAV or SARS-CoV-2 infection.

Diet-induced obesity also stimulates the proliferation of myeloid progenitors in murine bone marrow (171, 227). In a study by Christ et al. (171) using *Ldlr* ^−/−^ mice, a four-week WD induced granulocyte-monocyte-progenitor (GMP) proliferation and skewing toward development of activated monocytes, coupled with long-lived transcriptional and epigenetic reprogramming (171). Thus, obese mice on a WD not only have more immune cells, but they are ‘primed’ to release more inflammatory cytokines upon exposure to infectious stimuli than their non-obese counterparts.

### Initiation of Trained Immunity in Obesity

The immune stimuli that may induce trained immunity in obesity are yet to be comprehensively investigated. However, several factors associated with obesity may be implicated. As with the chronic low grade systemic inflammation triggered by a high fat diet, a dysregulated gut microbiota, in combination with ‘leaky gut’, results in increased circulating levels of LPS which could act as the primary inducer of innate immune training (228, 229). In addition, Oxidsed Low Density Lipoprotein (oxLDL), a marker of oxidative stress enhanced in obesity (230, 231), can induce a hyperinflammatory trained phenotype in human monocytes (232). Importantly, diabetes may further confound this. Diabetes and hyperglycemia induce intestinal barrier permeability, through transcriptional reprogramming of intestinal epithelial cells and disruption of junction integrity, which could contribute to enhanced circulating LPS (233). Furthermore, high glucose conditions have been shown to boost oxLDL training in monocytes compared to normoglycemic conditions (234), and induce enhanced proliferation of HSPCs in the bone marrow resulting in increased circulating myeloid cells (235). Bekkering et al. (236) discuss these factors in further detail, and speculate on a potential role of adipokines in immune training (237).

**Figure 2:**
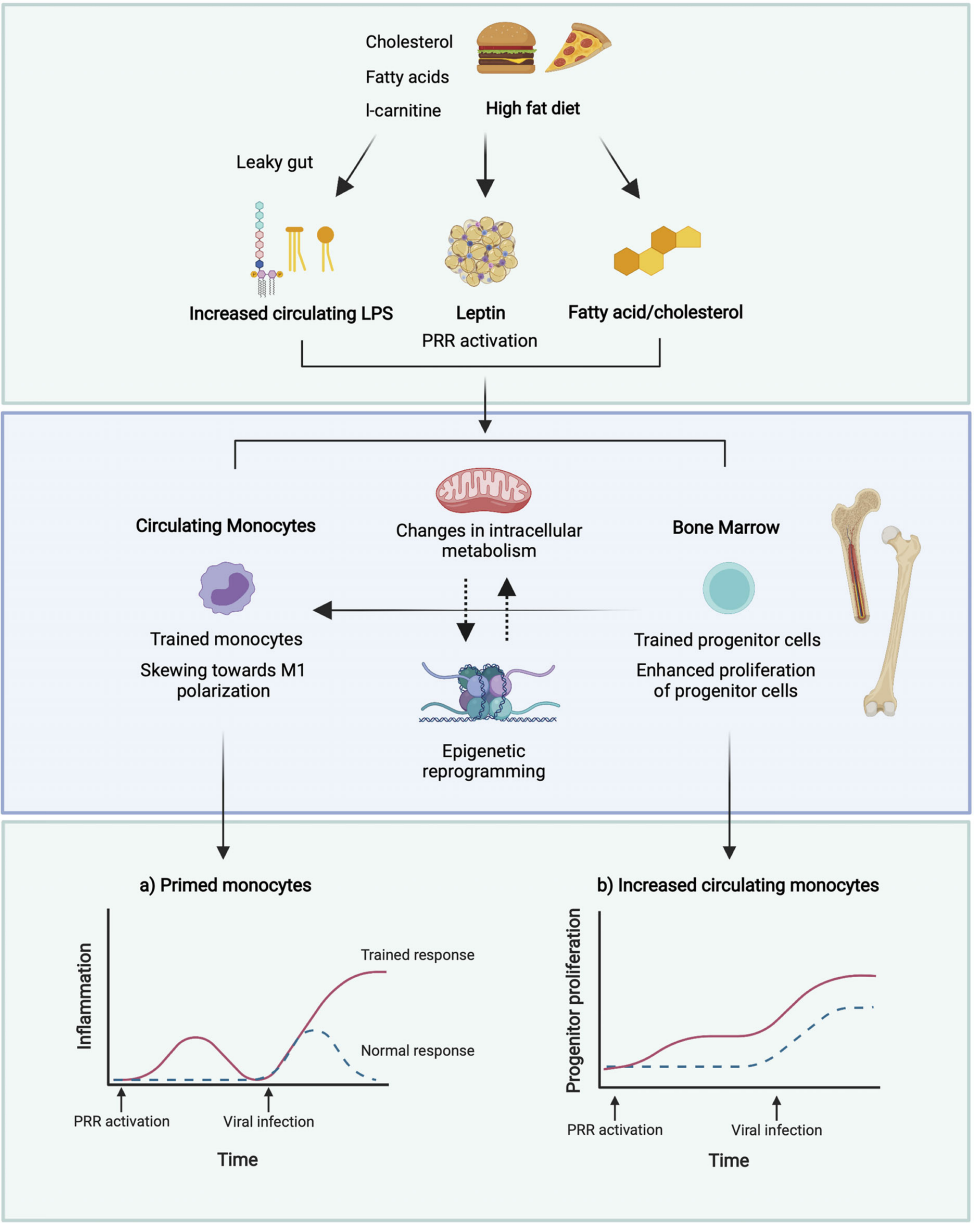
Potential mechanisms of induction of trained immunity in obesity. A leaky gut following a high dietary fat intake results in increased circulating levels of LPS (229) which may induce trained immunity (238, 239). High glucose conditions may boost immune training in monocytes and enhance the proliferation of HSPCs in the bone marrow (235). Reduced adiponectin in obesity may promote polarisation towards M1 macrophages and defective HSPC proliferation (240, 241), whilst increased leptin may promote proliferation and activation of circulating monocytes and BM myelopoiesis (242, 243). This may result in enhanced monocyte populations, and enhanced pro-inflammatory responses of these monocytes to viral infection. Figure created with BioRender.com.

Christ et al. (171) identified the NLRP3 inflammasome as the central receptor in immune reprogramming following a WD (171). IL-1β, as the best characterized inflammasome-dependent cytokine, may therefore be the central mediator behind the trained phenotype. Whether pharmaceutically targeting either NLRP3 or downstream cytokines can ‘reverse’ an obesity-induced WD phenotype, remains to be determined. The current hypothesis for how obesity drives meta-inflammation is detailed in **Figure 2**.

### Upsetting the Pro-Inflammatory/Anti-Viral Balance

The balance between pro-inflammatory cytokines and the antiviral IFNs must be tightly maintained, as these cytokines can negatively regulate one another (207). Age (244), genetics (107, 151) and obesity can skew this balance (245). Specifically, for IAV, Honce et al. showed that in normal human bronchial epithelial cells (NHBEC) derived from healthy or obese patients, there was a lower interferon response and increased viral replication in the obese NHBEC (245). Additionally, following infection with H1N1 (A/California/04/2009), obese mice had increased viral spread and increased viral diversity at 3 days post infection (d.p.i), despite similar viral titers to their wild type (WT) counterparts. This could be rescued with administration of recombinant IFN, suggesting that a blunted type I IFN response in obese mice drives this phenomenon (245). Whether this blunted IFN response was driven by increased pro-inflammatory cytokines in the baseline obese state was not determined (20). We speculate that similar experiments with SARS-CoV-2 will demonstrate that obesity blunts the anti-viral response to SARS-CoV-2, resulting in greater lethality. Pre-clinical studies of small molecules that stimulate an early IFN response show promise in animal models of COVID-19 (107, 246); treating obese patients who have been exposed to IAV or SARS-CoV-2 may help overcome the obesity-induced IFN deficit early in infection.

Considering how obesity increases HSPC proliferation and ‘primes’ these HSPCs through trained memory to produce more cytokines upon PRR stimulation, it is tempting to speculate that obesity-induced trained immunity drives pathological pro-inflammatory responses in IAV and SARS-CoV-2 infection. Curiously, the granulocyte-myeloid progenitors from WD- trained mice also had higher levels of type I IFN upon re-stimulation with TLR agonists (171). Based on this observation, a reasonable hypothesis would assume that WD training increases both IFN and pro-inflammatory cytokine release during infection, equating to quicker viral elimination and improved outcomes in the obese host, as the innate immune system is effectively ‘primed’ to fight infection. However, as described throughout this review, the opposite is true. This may reflect different cells being subjected to obesity-induced immune training, and distinct epigenetic reprogramming in different cells or tissues driving this skewed response. It could also reflect the cell-type specific viral tropism of IAV and SARS-CoV-2, and cell type specific pro-inflammatory or anti-viral cytokine release during infection. The specific cell types throughout the body, and particularly in the lung that are primarily ‘trained’ by obesity, and indeed, responsible for detrimental inflammation during IAV and SARS-CoV-2 infection, remain to be elucidated.

Consistent with obesity ‘training’ immune cells, PBMCs isolated from obese individuals had higher baseline levels of pro-inflammatory cytokines and reduced levels of anti-inflammatory cytokines compared to non-obese individuals (193, 194). Additionally, the PBMCs from the obese donors had elevated levels of the negative regulator SOCS3. Accordingly, these obese PBMCs had lower cytokine responses to *ex vivo* TLR3 and TLR7 stimulation. Elevated SOCS3 in obesity, because of meta-inflammation, may therefore blunt the anti-viral IFN response, rendering obese patients more susceptible to IAV or SARS-CoV-2 infection (247–249). As many PRRs and negative regulators of PRR signaling are themselves induced by cytokines or PAMPS and DAMPS, obesity may increase expression of PRRs, therefore sensitizing cells to viral PAMPs and DAMPS, while increasing expression of negative regulators such as SOCS3. If this occurs selectively, this may account for the decrease in anti-viral cytokines and concurrent increase in pro-inflammatory cytokines in obesity during viral infection.

### Impact on Adaptive Immune Responses

**Figure 3:**
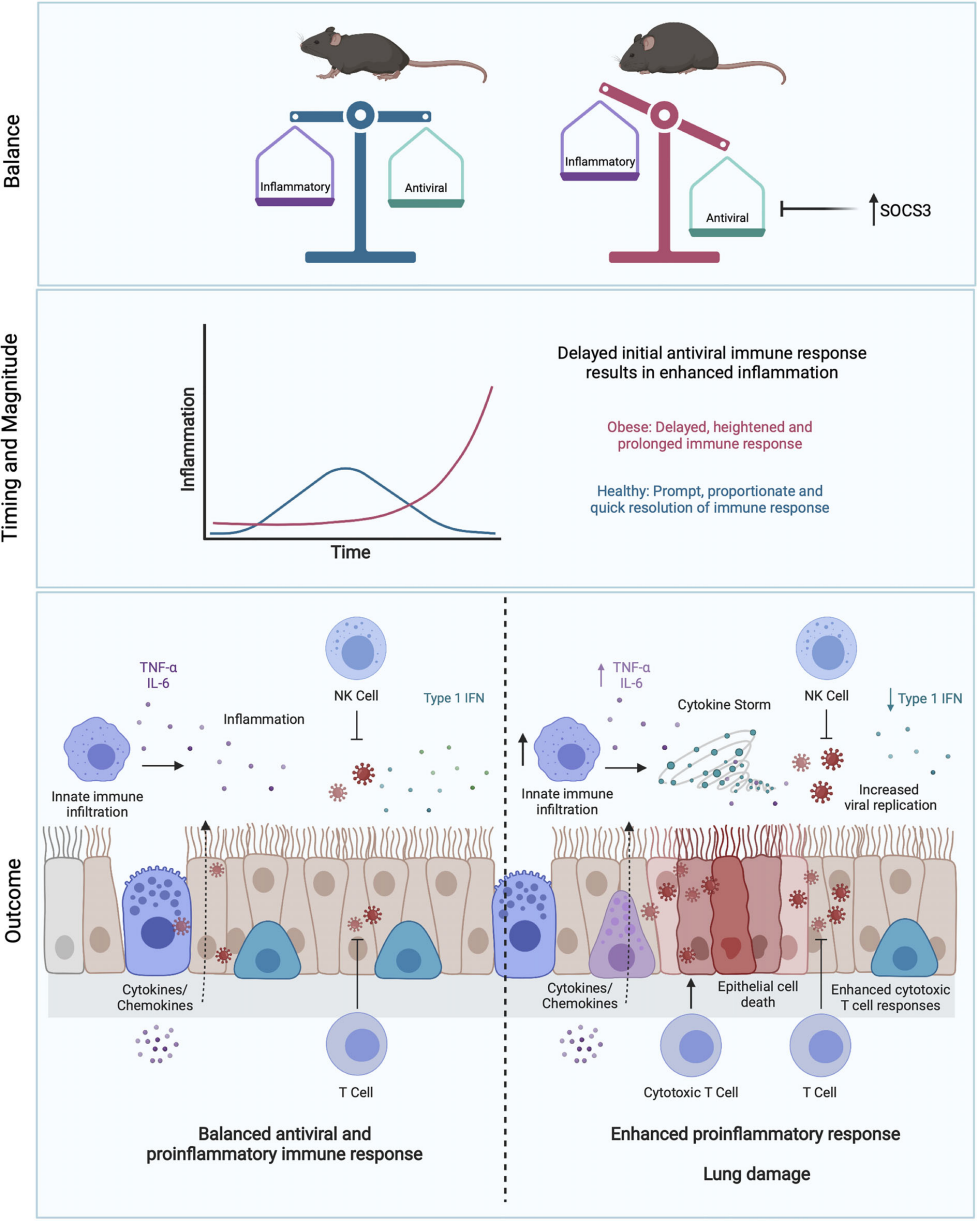
Obesity skews the balance between antiviral and pro-inflammatory responses. Initial blunted interferon responses to IAV in obese individuals results in increased viral spread and replication (245). This is accompanied by enhanced pro-inflammatory cytokine responses, driving immunopathology in the lung (193, 194). This imbalance may be partly driven by elevated levels of SOCS3 in obesity (193, 194). Delay of early viral clearance by T cells may result in further tissue damage due to excess CTL responses. Figure created with BioRender.com.

Obesity and obesity-associated inflammation also have a large impact on adaptive immunity, as reviewed by McLaughlin et al. (250). T and B cell responses are directed and potentiated by inflammatory cytokines (38), and it follows that any dysregulation in innate inflammatory responses will negatively impact adaptive responses. In an obese host, these inflammatory signals are delayed, but overall are increased during primary IAV infection (194), which could have the two-pronged effect of delaying viral elimination by T cells, then subsequently promoting tissue damage due to excessive CTL responses. Accordingly, minimal expression of IFN-α and IFN-β, along with a delayed pro-inflammatory response (specifically IL-6 and TNF) and reduced natural killer (NK) cell cytotoxicity in obese mice infected with IAV correlated with increased mortality (19). Obesity may also impair the maintenance of effector memory T cells in the lung. Following infection and clearance of X31 (H3N2), obese mice lost significantly more lung resident effector memory T cells compared to lean mice resulting in increased lung pathology, viral titers and mortality during a secondary PR8 (H1N1) infection (144). During this secondary infection, obese mice also had significantly less expression of IFN-γ in the lungs as well as significantly less IFN-γ-producing CD8+ T cells (144). This impairment of T cell responses in the obese state has also been linked to leptin resistance (144, 251–253). In COVID-19, lower CD4+ and CD8+ T cell counts were observed in patients with diabetes mellitus upon admission, which correlates with increased disease severity (254). Whether the same defects in T cell responsiveness in obese hosts are observed during SARS-CoV-2 infection, and whether this ultimately derives from the dysregulated inflammatory state, will be important to elucidate. In **Figure 3**, we propose how obesity drives disease severity in IAV and SARS-CoV-2 infection by dysregulating inflammation.

### Prevention of Disease Severity Through Non-Medical Interventions

Considering that obesity increases the risk of severe disease in viral respiratory infections, both medical and non-medical interventions to overcome this risk are desperately needed. Additionally, obesity decreases the efficacy of vaccination against IAV, making obese hosts more difficult to protect from severe IAV infection. Compared to non-obese counterparts, there is a greater reported decrease in influenza vaccine efficacy over time in the obese population, as well as reduction in the protective immune response (31, 255). Based on the suboptimal history of influenza vaccine efficacy in obese populations, there is understandably concern surrounding the efficacy of SARS-CoV-2 vaccines in these individuals. Currently it has been reported that the efficacy of a number of SARS-CoV-2 vaccines has been comparable between obese and non-obese participates (e.g., Pfizer-BioNTech, 95% efficacy overall, and 95.4% efficacy in obesity) (256). However, it remains to be seen if the efficacy of these vaccines wanes more quickly over time in obese populations compared to non-obese, similar to influenza vaccines. There is similarly limited evidence of the effectiveness of current influenza antiviral treatments in obese hosts (31), or efficacy of newly developed SARS-CoV-2 antivirals in the general population (257), let alone the obese population.

Given the challenges in directly targeting these viruses with vaccination and anti-virals in both the general population and in obese risk groups, dietary changes, exercise and both surgical and non-surgical weight loss strategies all need to be examined as measures to reverse obesity and treat its associated co-morbidities.

### Surgical and Non-Surgical Weight Loss

Weight loss is an essential intervention in obesity that reduces the severity of many of its non-communicable complications (diabetes mellitus, pulmonary disease, cardiovascular disease, etc.) (5). Bariatric and metabolic surgery (BMS) (including gastric bypass, adjustable gastric banding and sleeve gastrectomy) is a proven treatment for obesity (258). BMS reduced the risk of all-cause mortality and ensured patients live longer than non-surgical matched controls (259–265). BMS reduces the inflammation of adipose tissue, specifically by downregulating HIF-1α, PLAUR, MCP-1 and CSF-3 gene expression and thus dampens the infiltration of macrophages into adipose tissue, and enhances expression of anti-inflammatory cytokines such as IL-10 in the remaining adipose tissue macrophages (266). BMS also reduces the level of circulating CD4+ and CD8+ T cells and leptin (267), which may protect from T-cell induced immunopathology. As little as 6 months post-operative, BMS reduced serum concentrations of inflammatory cytokines including CRP, IL-1β, IL-6, IL-8, IFN-γ, IL-12, IL-23, and TNF (268).

To date, the only study specifically investigating the effect of weight loss in IAV infection has focused on the memory T-cell response (269). Rebeles and colleagues demonstrated that obese mice have impaired T-cell metabolism and memory T-cell responses to IAV and that this impaired response is maintained, even after weight loss (269).

Christ et al. observed that innate immune cells from obese mice were hyperinflammatory, and that this hyperinflammatory state was maintained even if the obese mice had lost weight (171). Whether this trained immune phenotype observed in previously obese mice is also detrimental in primary influenza infection remains to be determined. However, we speculate that formerly obese patients may still be at risk of developing severe influenza even if they have lost weight, due to the legacy of trained immunity driving a baseline hyperinflammatory response.

Clinical evidence of the long-term effects of obesity on influenza and COVID-19 is still scarce. Most often, these clinical studies refer to respiratory infections or influenza-like illness (ILI) without laboratory confirmation. One such retrospective study looking at ILI focused on the 2018-2019 influenza season in France and Italy (270). This study compared the outcomes of obese non-surgery (NS) patients and BMS patients. When compared to the obese NS patients, BMS patients reported significantly lower incidence of fever and ILI, lower intake of non-steroidal anti-inflammatory drugs, and lower admittance to the emergency department for ILI. While this study demonstrates a lower incidence of ILI in BMS patients compared to NS obese patients, this retrospective study did not compare BMS patients to the general non-obese population. In a retrospective, population-based, matched cohort study spanning 2005-2013 in Taiwan, significantly lower risk of respiratory tract infection (RTI), including influenza, and shorter hospital stays were reported in the BMS group compared to the NS (271). When compared to the general population however, BMS patients were still at a significantly higher risk of RTI and specifically influenza (271). Whether these BMS patients still have obesity-associated meta-inflammation and impaired interferon or adaptive responses, remains to be elucidated.

Weight loss and exercise studies in both humans and animal models are often quite long term. Due to the relative recency of SARS-CoV-2 appearing in the human population, research into the effect of weight loss in this context is severely limited. Furthermore, due to the current load on the health care system and the intraoperative risks for viral contagion among patients and staff due to COVID-19, many centers have postponed all elective surgeries including BMS (258, 265). As such, there are no long-term studies investigating whether BMS reduces the risk of COVID-19 compared to NS. To date, there has been one study in London that observed that over a two month period during a peak COVID-19 period, only one out of a total of 1439 patients presenting with COVID-19 had previously undergone BMS (258). This study tentatively demonstrates that BMS does not increase the risk of COVID-19 infection compared with the general population (258). However, the study did not report on what percent of the cohort were obese.

Together, these experimental and retrospective studies suggest that weight loss through both surgical and non-surgical means reduce the risk of RTI when compared to obese individuals. However, when compared to individuals with no history of obesity, those who underwent weight loss were still at a higher risk of infection and severe outcomes. This indicates that there are indeed long-term effects of obesity on the immune system that cannot be reversed with weight-loss alone, and pre-dispose these formerly obese individuals to higher risk of severe disease during IAV or SARS-CoV-2 infection. Of note, none of the studies reported on the exercise habits of participants, which could also affect outcomes. A summary of these interventions is detailed in **Table 2**.

**Table 2:** Medical and non-medical interventions to reverse long-term immune effects of obesity.

Obesity Model (Murine or Human)	Intervention	Disease Model	Outcome	Mechanism	Reference
***General Studies***					
Murine (HFD)	Voluntary Exercise	Cardiovascular inflammation	Lower leptin, leukocytes, myeloid cells, lymphocytes, neutrophils, B cells, T cells, monocytes, and overall inflammation	Leptin regulation of HSPC proliferation and leukocyte production in bone marrow	(272)
Clinical	BMS	Obesity-driven inflammation	Reduced the inflammation of adipose tissue and dampened the infiltration of macrophages into adipose tissue	Downregulation of HIF-1α, PLAUR, MCP-1 and CSF-3 reduced macrophage recruitment in subcutaneous white adipose tissue	(266)
Clinical	BMS	Obesity-driven inflammation	Reduces the level of circulating CD4+ and CD8+ T cells and leptin	Leptin regulation of leukocyte production	(267)
Clinical	BMS	Obesity-driven inflammation	Three months after surgery there is reduced levels of insulin, HOMA-IR and lipid parameters. Also a reduction in macrophage infiltration to adipose tissue and reduced crown-like structure (CLS) density Six months after surgery there is reduced serum concentrations of inflammatory cytokines including CRP, IL-1β, IL-6, IL-8, IFN-γ, IL-12, IL-23, and TNF.	Reduction in adipocyte hypertrophy after BMS, leads to reduced CLS density and thus downstream reduction in systemic inflammation	(268)
Clinical	Voluntary Exercise	Obesity-driven inflammation	Reduced circulating leptin and leukocyte levels	Leptin regulation of HSPC proliferation and leukocyte production in bone marrow	(273)
***Influenza Specific Studies***					
Murine (HFD)	Diet-induced weight loss (after primary infection)	Influenza A X-31 H3N2 (Primary) PR8 H1N1 (Secondary)	Weight loss reversed systemic hyperinsulinemia, hyperglycemia and reduced visceral adipose tissue mass However obesity associated impaired memory T-cell response and metabolism were maintained after weight loss	Obesity-associated T-cell metabolic dysfunction	(269)
Retrospective Clinical Study	BMS	Influenza-like Illness	Compared to NS: - Lower incidence of fever and ILI - Less likely to be admitted to ED for ILI	Unknown	(270)
Retrospective Clinical Study	BMS	Influenza-like Illness	Compared to NS: - Lower risk of RTI and Influenza - Shorter hospital stays Compared to general population: - Significantly higher risk of RTI and influenza	Unknown	(271)
Retrospective Clinical Study	Voluntary Exercise	Influenza	Compared to never or seldom exercise: - Lower risk of influenza-associated mortality	Unknown	(274)
Murine (non-obese model) Non-obese model	Exercise (during IAV infection)	H1N1 Influenza A (A/Puerto Rico/8/34)	Moderate exercise: - Decreased mortality post infection (18%) compared to sedentary (56%) Prolonged exercise: - Increased mortality post infection (70%) compared to sedentary (56%)	- Less cellular infiltration and IFN-γ in the lungs, in addition to a shift to Th2 response - Shift to Th2 response	(275)
Murine (HFD)	Exercise (prior to IAV infection)	H1N1 Influenza A (A/Puerto Rico/8/34)	- Reduced TNF-α production by influenza specific CD8+ T cells - Increased serum anti-influenza virus specific IgG2c antibody levels - Increased IFN-α related gene expression	Restore type I interferon response	(276)
Murine (HFD)	Vitamin A supplementation	pdmH1N1 Influenza A (A/California/04/2009 pdmH1N1)	- Reduced viral loads - Improved antibody response to IAV	Reduced circulating inflammatory cytokines	(277)
***COVID-19 Specific Studies***					
Retrospective Clinical Study	BMS	SARS-CoV-2	1/1439 patients presenting with COVID-19 had BMS	Unknown	(258)

### Exercise

Independent of surgical and non-surgical weight loss, exercise also has anti-inflammatory effects. Voluntary exercise lowered leptin levels in mice fed HFD and was accompanied by a drop in total numbers of leukocytes, lymphocytes, neutrophils, B cells, T cells, monocytes, and overall chronic inflammatory markers (272).

Exercise also reduced circulating leptin and leukocyte levels in a cohort of Japanese adolescent males (273). Additionally, low to moderate frequency exercise was associated with a lowered risk of influenza-associated mortality compared to those who never or seldom exercise (274). However, neither of these studies reported the BMI of participants (274), making it difficult to interpret the efficacy of exercise in lowering obesity-associated in these populations.

In both obese and non-obese mice, exercise increased serum anti-influenza virus specific antibodies, T cells and improved disease outcomes (276). This correlated with increased chemokine, cytokine and importantly IFN expression in both groups post-exercise treatment. Thus, exercise may restore the obesity-associated pro-inflammatory cytokine and Type I interferon imbalance to improve disease outcomes, though the exact mechanisms still need to be elucidated.

Collectively, this evidence suggests that independent of other interventions, voluntary moderate physical activity alone both prior to- and during infection can improve infection outcomes. With demonstrated benefits of exercise for both obese and non-obese individuals, engaging in at least 150 minutes of moderate aerobic exercise each week for the general population may be a useful strategy to improve viral respiratory disease outcomes (278).

### Dietary Changes/Supplementation

There are numerous animal and human studies that support the health benefits of intermittent fasting, including weight loss, improved glucose tolerance and insulin sensitivity, and reduced circulating and adipose levels of inflammatory biomarkers (255). Intermittent fasting could therefore offer an avenue for improving immune system function without drastic lifestyle changes. Notably, intermittent fasting reduces circulating and adipose levels of leptin, IL-6, TNF, IGF-1, IL-1β, and CRP (255, 279). While there are currently no studies investigating the effect of intermittent fasting on influenza or COVID-19 severity, it has been suggested as a possible non-medical prophylactic tool for both obese and non-obese individuals, and warrants further investigation (255, 280).

In addition to elevated fat stores and meta-inflammation, individuals with obesity also suffer from tissue-specific vitamin A deficiency that is often masked due to normal serum retinol levels (277, 281). Vitamin A is essential for hematopoiesis and both innate and adaptive immune function (282) and as such it has been suggested vitamin A deficiency could play a role in the severity of viral infections and the poor outcome often associated with vaccination in obese individuals (277). In a murine model of obesity with IAV vaccination and challenge, vitamin A supplementation decreased viral loads, reduced circulating inflammatory cytokines and improved antibody responses (277). Vitamin A may therefore be a low-cost intervention to increase vaccine efficacy and reduce IAV and SARS-CoV-2 disease severity in obese individuals.

### Medical Interventions to Treat Obesity Induced-Inflammation

A variety of analogues and mimetics for FGF21 and GDF15 have been investigated for treatment of obesity. Clinical trials for FGF21 have been comprehensively detailed in a review by Geng et al. (236). In brief, long-term treatment with hFGF21 analogues have been shown to reduce dyslipidaemia, enhance insulin sensitivity and reduce body weight in clinical trials (236). In addition, preclinical obesity studies have shown treatment with GDF15 decreases food intake and body weight (283). However, the benefit of augmented FGF21 and GDF15 treatment in reducing risk for severe IAV and SARS-CoV-2 infection in obesity has not yet been explored.

While directly targeting inflammation with anti-inflammatory treatments is beneficial in COVID-19, there are limited studies on the efficacy of anti-inflammatories in decreasing IAV or SARS-CoV-2 disease severity in obesity. Tocilizumab (which blocks IL-6 signaling) was administered to treat the hyperinflammation of critical COVID-19 obese patients during the first days of worsening hypoxemia. In a case series, three obese patients from different age groups with COVID-19 were treated with Tocilizumab. All patients showed improved clinical signs and were discharged from hospital without complications or intubation, even when the ideal dose of infusion was not feasible to be administered (284). Randomized controlled trials of anti-inflammatories such as dexamethasone or tocilizumab in obese patients with IAV and COVID-19 will further determine if suppressing baseline inflammation is beneficial.

A pre-clinical anti-inflammatory drug that shows promise is 5-deoxy-Δ12, 14-prostaglandin J2 (15d-PGJ2), an anti-inflammatory lipid mediator and peroxisome proliferator-activated receptor-gamma (PPAR-γ) agonist. 15d-PGJ2 has proved beneficial in inhibiting melanoma progression in cancer (285), and reducing lung inflammation and remodeling in asthma (286) by inhibiting NF-κB signaling. In obese mice (*db/db*), PPAR-γ, an important inflammation regulator, is downregulated in lung macrophages following IAV infection. Treatment with 15d-PGJ2 improved the overall survival of IAV-infected obese mice, and was attributed to its effect on macrophage function (287). As of yet, there are no clinical studies using 15d-PGJ2 for either IAV or SARS-CoV-2.

Metformin is another commonly prescribed, clinically approved drug that is a possible candidate for repurposing to treat obesity and prevent IAV and SARS-CoV-2 disease severity. Metformin decreases mitochondrial oxidation and is primarily prescribed as a drug to treat Type 2 Diabetes. Metformin also improved the survival of obese mice in IAV infection (288). Mechanistically, this was attributed to metformin normalizing CD4+ T cell glucose oxidation and therefore function in the obese mice, though it is likely metformin has a positive effect on multiple immune cell populations (288). Interestingly, this study compared metformin treatment to a weight loss treatment group, which was not sufficient to reverse the obesity-related effects on CD4+ T cells, or improve survival, suggesting long lasting immune alterations. While metformin has not been used in the clinic to treat severe IAV infection, there have now been clinical studies targeting COVID-19. Metformin reportedly reduces the mortality in women with obesity or Type 2 Diabetes who were admitted to hospital with COVID-19 (254, 289).

In addition to experimental and prescription drugs, there is evidence that that non-steroidal anti-inflammatory drugs (NSAIDs), which are often easily accessible over the counter without a prescription, could prove beneficial for treating hyperinflammation in obesity and severe viral infections. NSAIDs have previously been associated with adverse clinical outcomes in the context of bacterial community-acquired pneumonia (290, 291), and as such there has been hesitation to use NSAIDs in the context of influenza and COVID-19. However, the use of NSAIDs was not associated with adverse outcomes (e.g. hospitalization, ICU admission, mechanical ventilation, mortality) in the context of the 2009 IAV pandemic (292), seasonal influenza (293), and the SARS-CoV-2 pandemic (294, 295). Despite NSAIDs targeting components of the cyclooxygenase (COX) pathway, which are hyper-induced during influenza infection (110, 296, 297), there is limited research into their potentially beneficial effects in the context of influenza and COVID-19. NSAIDs inhibit the inflammatory prostaglandin (lipid mediator) synthesis pathway by targeting the key enzyme cyclo-oxygenase-2 (COX-2) (298). Mice deficient in COX-2 had reduced mortality and viral titer after IAV infection, despite their blunted inflammatory response (299). Targeting specific inflammatory prostaglandins, rather than the central enzyme COX-2, also shows promise in treating severe IAV. Prostaglandin E2 (PGE2) inhibition improved survival after a lethal dose of IAV in a mouse model (300). PGE2 itself is upregulated during IAV infection and inhibits host type I IFN responses, macrophage recruitment to the lungs, and potentially impairs antigen presentation and T-cell mediated immunity (300). Whether broadly targeting prostaglandins *via* COX-2 inhibition or selectively targeting individual prostaglandins such as PGE2 improves outcomes in obesity and viral infection should be further examined.

While there is no data yet on NSAIDs in SARS-CoV-2 outcomes, there is currently a phase IV randomized clinical trial underway in the U.K., aimed at evaluating use of lipid ibuprofen in the reduction in severity and progression of SARS-CoV-2 infections (301). Whether NSAIDs further benefit the obese host in IAV and SARS-CoV-2 infections still needs to be determined. Future research might also include combination therapy of anti-virals with anti-inflammatories specifically in obese patients, which could augment pathogen elimination while suppressing detrimental inflammation, and in effect, correcting the dysregulated immune response.

### Conclusions and Future Directions

Treating severe respiratory viral infections, particularly in a pandemic context, requires fundamental understanding of the mechanisms of disease pathology. Here we have reviewed how inflammation is initiated in IAV and SARS-CoV-2 infection, and how dysregulated inflammation can drive disease severity. Obesity is a key co-morbidity that profoundly impacts the immune response to these viral infections. While the molecular mechanisms by which diet induces a meta-inflamed state are increasingly well understood, some outstanding questions remain. How does obesity modulate the specific cell types and PRR signaling pathways involved in IAV and SARS-CoV-2? How do protective inflammatory responses become detrimental during IAV or SARS-CoV-2 infection? In answering these questions we may be able to identify further critical regulators of the inflammatory response (e.g., SOCS3) that are dysregulated in obesity and other co-morbidities. Identifying new and selective drug targets that preferentially suppress detrimental inflammatory responses while preserving anti-microbial responses will benefit both obese and non-obese individuals.

As our understanding of obesity and dietary-induced inflammation broadens, it is becoming increasingly apparent that diet-induced meta-inflammation is not limited to obese hosts. Multiple studies now suggest that a history of obesity is enough to confer a meta-inflamed state, and that this may pre-dispose these individuals to a higher risk of severe IAV or SARS-CoV-2 infection. Further research is warranted to understand the mechanisms of obesity-induced training, whether a trained phenotype lasts after weight loss, and if so, for how long. In addition, the key cell types that undergo obesity- induced training have yet to be identified. Cells in the mucosal periphery, such as alveolar macrophages (AMs) in the lung, have recently been shown to be subject to training independently of the bone marrow (302). This is particularly important in the case of viral infection; AMs reside in the epithelial fluid and are distinct from macrophages residing between the airway epithelium and blood vessels (303). As such, they are the first in line of defense against an influenza infection (304). As only 40% are replaced yearly, the lifespan of AMs could result in long lasting changes in the innate immune reponse in the lung (304, 305). The presence of immune training in these cells following a period of obesity has yet to be investigated. Similarly, whether diet-induced long-term immune training can be reversed is still unknown. This is of particular importance given that BMS and weight loss alone is insufficient to reduce the risk of developing influenza, and previously obese individuals still remain at a higher risk of RTIs compared to the general population (271).

In conclusion, while obesity is a multifactorial disorder with multiple effects on the host response to infection, a key mechanism by which obesity increases virus induced disease severity is by dysregulating the inflammatory response. Obesity primes the innate immune system to respond to IAV and SARS-CoV-2 with a heightened pro-inflammatory response and a blunted anti-viral response, which ultimately leads to increased tissue damage and decreased virus elimination. Together, this drives the increased disease severity observed in obese hosts in both influenza and COVID-19.

## Author Contributions

Conceptualization: KH, KS, and LL. Original drafts: KH, EN, and LL. Figures: EN. Editing: KH, EN, KS, and LL, Supervision: KS and LL. All authors contributed to the article and approved the submitted version.

## Funding

This work is supported by the National Health and Medical Research Council of Australia (Fellowship 1124162 to LIL), the Australian Research Council (Fellowship DE180100512 to KS) and a UQ Early Career Researcher Grant (UQECR2058045) to LL.

## Conflict of Interest

The authors declare that the research was conducted in the absence of any commercial or financial relationships that could be construed as a potential conflict of interest.

## Publisher’s Note

All claims expressed in this article are solely those of the authors and do not necessarily represent those of their affiliated organizations, or those of the publisher, the editors and the reviewers. Any product that may be evaluated in this article, or claim that may be made by its manufacturer, is not guaranteed or endorsed by the publisher.
